# Can monolithic zirconia frameworks in implant-supported cross-arch prostheses deliver reliable long-term outcomes?

**DOI:** 10.1038/s41432-025-01108-9

**Published:** 2025-01-29

**Authors:** Nidhi Parmar

**Affiliations:** Eastman Dental Institute, London, UK

**Keywords:** Fixed prosthodontics, Dental implants, Dental implants, Dental treatment planning, Dental biomaterials

## Abstract

**Design:**

A retrospective cohort study assessing the mid-to-long-term outcomes and risk factors affecting the prosthetic success and survival of implant-supported cross-arch fixed dental prostheses (IFCDPs) with monolithic zirconia frameworks.

**Cohort selection:**

Forty-seven patients received a total of 51 cross-arch prostheses (27 maxillary and 24 mandibular prostheses), supported by 302 implants. Comprehensive clinical and radiographic records were available over a follow-up period ranging from 5 to 13 years. A strict inclusion criteria ensured the use of screw-retained implants and monolithic zirconia frameworks fabricated using standardised CAD/CAM protocols, without cemented titanium bases. Exclusion criteria included systemic conditions affecting healing, bruxism, uncontrolled periodontitis, smoking, and significant health changes during the follow-up period.

**Data analysis:**

Descriptive statistics summarised implant and prosthesis outcomes, while complications were evaluated for peri-implantitis at an implant level and framework fractures at a prosthesis level. Peri-implantitis was identified through clinical signs, including bleeding on probing, suppuration, and radiographic evidence of bone loss. Prosthetic outcomes were classified using the modified USPHS criteria. Mixed-effects Cox regression models were applied to analyse risk factors. Hazard ratios were calculated for peri-implantitis and framework fractures, with statistical significance set at *p* < 0.05.

**Results:**

The implant survival rate was 97.64%, with peri-implantitis observed in 27 implants, predominantly in the mandible, resulting in an overall implant success rate of 91.06%. Prosthesis survival was 82.35%, with nine framework fractures reported, eight of which occurred in mandibular prostheses. The mandible was identified as a significant risk factor for both framework fractures (HR = 11.64, *p* = 0.024) and peri-implantitis (HR = 10.88, *p* = 0.003).

**Conclusion:**

IFCDPs with monolithic zirconia-based frameworks exhibited favourable clinical outcomes over a 5–13-year period. However, mandibular prostheses were more prone to framework fractures and peri-implantitis, highlighting the need to consider mandibular flexure in prosthetic design to enhance long-term success and durability.

## A Commentary on

**Luo J, Zhang Y, Yu Z** **et al**.

A retrospective single cohort study on the 5–13-year clinical outcomes of implant-supported cross-arch fixed dental prostheses with monolithic zirconia-based frameworks. *J Prosthodont* 2024; 10.1111/jopr.13991.

**GRADE Rating**: Medium 

## Commentary

The 2018 ITI Consensus Report highlights the potential of implant-supported monolithic zirconia prostheses as a promising option, subject to further supporting evidence^1^. Zirconia frameworks show promising short-term outcomes, with survival rates for full-arch zirconia rehabilitations ranging from 88% to 100% at 7 years^2^. However, comprehensive analyses of mid-to-long-term clinical performance of screw-retained monolithic zirconia IFCDPs and associated risk factors remain limited^3^.

The study’s strengths lie in its comprehensive evaluation of both biological (peri-implantitis) and technical complications (framework fractures), offering a holistic view of the performance of zirconia-based IFCDPs^4^. Longitudinal data over 5–13 years, offer rare insights into prosthesis survival and the progression of complications, addressing a significant gap in existing literature. Based on the CASP checklist, the study had clear aims, employed an appropriate retrospective methodology following STROBE guidelines, measured outcomes using standardised records and USPHS criteria. The use of mixed-effects Cox regression models to assess correlations between clinical variables and complications, enhances statistical rigour. The analysis accounted for clustering effects from multiple implants or prostheses per patient and evaluated variables such as jaw type, implant count, cantilever presence, and opposing dentition.

Whilst the findings of this study reinforce the viability of monolithic zirconia frameworks, it also highlights specific challenges, underscoring the complex interplay between material properties and anatomical factors. The natural flexure of the mandible generates bending stresses, resulting in deformation during mandibular movement, particularly in the symphyseal region. It has been suggested that the non-ductile nature of monolithic zirconia may insufficiently resist these stresses, resulting in stress concentration at critical points, such as the osseointegration interface of implants and the connection interface between implants and the superstructure. Clinically, the study’s identification of mandibular flexure as a significant risk factor for framework fractures and peri-implantitis, challenges assumptions of uniform performance across jaws. In turn, highlighting the importance of anatomical considerations in treatment planning. The identification of mandibular flexure as a risk factor emphasises the need for customised prosthetic designs, such as segmented frameworks or those incorporating titanium bases, to mitigate stress concentrations. These insights provide actionable guidance for pre-surgical planning and patient-specific treatment strategies, enhancing long-term outcomes.

Despite its strengths, the study has notable limitations. The retrospective cohort design restricts causal interpretation and depends on existing records, making it vulnerable to selection bias and incomplete data. The single-centre recruitment process and relatively small sample size, limits the generalisability and statistical power of subgroup analyses, particularly for maxillary versus mandibular outcomes. Furthermore, by not accounting for confounding variables such as oral hygiene factors, a well-established risk for peri-implantitis, limits a comprehensive understanding of complication dynamics. Additionally, the study does not explore alternative materials or designs in depth, leaving an opportunity for future comparative studies to examine options like titanium-based or hybrid frameworks.

To conclude, this study contributes valuable long-term data on monolithic zirconia frameworks in IFCDPs. It provides a foundation for innovation in material science and prosthetic design, paving the way for more tailored and durable solutions in implant dentistry.

Practice points

- This study contributes to the growing body of evidence supporting the use of monolithic zirconia frameworks in IFCDPs while also highlighting their limitations.
- By demonstrating that mandibular prostheses are particularly susceptible to mechanical and biological complications, it provides a nuanced understanding that can guide treatment planning.
- Moreover, the findings have implications beyond implant dentistry, as they contribute to the broader understanding of how anatomical and material factors influence prosthetic outcomes.

## References

[1] Morton D, Gallucci G, Lin WS, Pjetursson B, Polido W, Roehling S, et al. Group 2 ITI consensus report: prosthodontics and implant dentistry. Clin Oral Implants Res. 2018;29:215–23. 30328196 10.1111/clr.13298

[2] Cinquini C, Alfonsi F, Marchio V, Gallo F, Zingari F, Bolzoni AR, et al. The use of zirconia for implant-supported fixed complete dental prostheses: a narrative review. Dent J. 2023;11:144. 10.3390/dj11060144 PMC10297443 37366667

[3] Delucchi F, De Giovanni E, Pesce P, Bagnasco F, Pera F, Baldi D, et al. Framework materials for full-arch implant-supported rehabilitations: a systematic review of clinical studies. Materials. 2021;14:3251. 34204681 10.3390/ma14123251 PMC8231547

[4] Luo J, Zhang Y, Yu Z, Jiang X, Li J, Chen B, et al. A retrospective single cohort study on the 5-13 year clinical outcomes of implant-supported cross-arch fixed dental prostheses with monolithic zirconia-based frameworks. J Prosthodont. 2024. 10.1111/jopr.13991. 10.1111/jopr.13991 39655784

